# Indoor spraying with chlorfenapyr (a pyrrole insecticide) provides residual control of pyrethroid-resistant malaria vectors in southern Benin

**DOI:** 10.1186/s12936-020-03325-2

**Published:** 2020-07-13

**Authors:** Corine Ngufor, Augustin Fongnikin, Neil Hobbs, Martial Gbegbo, Laurette Kiki, Abibath Odjo, Martin Akogbeto, Mark Rowland

**Affiliations:** 1 London School of Hygiene and Tropical Medicine (LSHTM), London, UK; 2 Centre de Recherches Entomologiques de Cotonou (CREC), Cotonou, Benin; 3 Panafrican Malaria Vector Research Consortium (PAMVERC), Cotonou, Benin

**Keywords:** Experimental huts, Chlorfenapyr, Mixtures, Indoor residual spraying, Alpha-cypermethrin, Bendiocarb, Sylando, CDC Bottle bioassays, Anopheles, Cové

## Abstract

**Background:**

New classes of insecticides with novel modes of action, which can provide effective and prolonged control of insecticide-resistant malaria vector populations, are urgently needed for indoor residual spraying. Such insecticides can be included in a rotation plan to manage and prevent further development of resistance in mosquito vectors of malaria. Chlorfenapyr, a novel pyrrole insecticide with a unique mode of action, is being developed as a long-lasting IRS formulation.

**Methods:**

The efficacy of several formulations of chlorfenapyr alone and as mixtures with alpha-cypermethrin were evaluated in an experimental hut trial against wild pyrethroid-resistant *Anopheles gambiae* sensu lato in Cové, Benin, in an attempt to identify the most effective and long-lasting formulations for IRS. The trial lasted 12 months. A comparison was made with alpha-cypermethrin and bendiocarb formulations. CDC bottle bioassays were performed to investigate cross-resistance to chlorfenapyr in the local vector population.

**Results:**

Mortality rates in World Health Organization (WHO) cylinder bioassays were < 5% with pyrethroids due to high levels of pyrethroid resistance, but > 95% with bendiocarb thus confirming susceptibility to carbamates in the vector population. CDC bottle bioassays showed no cross-resistance between pyrethroids and chlorfenapyr. Overall mortality of free-flying mosquitoes entering the experimental huts over the 12-month trial was 4% with alpha-cypermethrin and 12% with bendiocarb. The chlorfenapyr solo-formulations induced significantly higher levels of mortality (38–46%) compared to the bendiocarb (12% P < 0.001) and to the mixture formulations (18–22%, P < 0.05). The original Sylando 240SC formulation of chlorfenapyr was more efficacious than all other novel chlorfenapyr formulations tested. Bendiocarb induced > 80% mortality in the first month, but this declined sharply to < 20% by the third month while the mortality rates achieved with the chlorfenapyr formulations (38–46%) were persistent lasting 7–10 months. The mixtures induced significantly lower percentage mortality than chlorfenapyr-solo formulations. Wall cone bioassays only showed mortality rates that were consistent with chlorfenapyr IRS treated huts when the exposure time was increased to 2 h.

**Conclusion:**

Indoor residual spraying with chlorfenapyr (Sylando^®^ 240SC) provides moderate but prolonged control of pyrethroid-resistant malaria vectors compared to pyrethroid and bendiocarb IRS. Wall cone bioassays on chlorfenapyr-treated walls required longer exposure times of 2 h than the customary 30 min indicating that WHO guidelines on residual cone bioassays need to be more insecticide-specific.

## Background

Indoor residual spraying (IRS) is a key vector control intervention for preventing or controlling malaria in many malaria-endemic countries [1]. When applied correctly, IRS can rapidly reduce vector populations and interrupt transmission. The use of IRS has increased considerably over the past two decades [2] contributing significantly to the remarkable reductions in malaria burden observed in endemic countries over this period.

The effectiveness of IRS for vector control depends on the residual activity of the insecticide on treated home walls and the continued susceptibility of local vectors to the insecticides being deployed. Until very recently, this intervention relied on a small number of insecticides from four classes of chemistry (pyrethroids, organophosphates, carbamates and the organochlorine DDT). Most of the insecticides also showed low residual activity (2–5 months) on wall substrates, thus requiring multiple rounds of IRS application for effective control [3]. Unfortunately, malaria vectors have developed resistance to all four classes of compounds [4, 5]. Resistance to pyrethroids and DDT is now widespread and increasing in intensity the more they are used, making these compounds less effective as time goes on [5]. This resulted in increased use of carbamates and organophosphates in recent years [2, 6] driven by the development of a long-lasting formulation of pirimiphos-methyl (Actellic^®^ 300CS) [7]. However, carbamate and organophosphate resistance is also increasing in malaria vectors especially in West Africa [4, 5, 8, 9].

The development of new IRS insecticides which can provide improved and prolonged control of insecticide-resistant vector populations is critical for maintaining the effectiveness of this intervention. Two new IRS insecticide products belonging to the neonicotinoid class have very recently been approved by the World Health Organization (WHO) [10]. As a strategy for managing insecticide resistance in local vector populations, vector control programmes are encouraged to implement a rotational schedule of IRS insecticides of different modes of action to reduce selection pressure on the vector [11]. While the newly prequalified IRS insecticides show potential to improve the control of insecticide-resistant vector populations [12–15], an effective rotational strategy which will prevent the development of further resistance cannot rely on these compounds alone as they contain the same active ingredient (clothianidin) and thus share the same mode of action; a more diversified portfolio of IRS insecticides is required.

Chlorfenapyr, a pyrrole insecticide has shown potential to control pyrethroid-resistant *Anopheles gambiae*. It works by targeting the oxidative pathways in the insect’s mitochondria disrupting ATP production and thus presenting a novel mode of action for IRS which differs from other public health insecticides [16–18]. There are currently no records of cross-resistance to chlorfenapyr and existing IRS insecticides [18]. While experimental hut trials with one formulation of chlorfenapyr for IRS (Sylando^®^ 240SC) have reported comparable mortality rates against wild free-flying pyrethroid-resistant malaria mosquitoes to some conventional IRS products [17], reformulation of insecticides can the increase residual activity of many classes of IRS insecticides such as pyrethroids [19] and organophosphates [7] and improve bioavailability and efficacy on treated wall substrates.

In this study, the bioefficacy of a series of new formulations of chlorfenapyr and mixtures of chlorfenapyr with alpha-cypermethrin for IRS against wild, free-flying pyrethroid-resistant *An. gambiae* sensu lato (*s.l*.) was assessed in experimental huts. To provide a more comprehensive understanding of the bioefficacy and residual activity of chlorfenapyr, the trial was conducted for 12 months and comparison was made with IRS with alpha-cypermethrin and bendiocarb.

## Methods

### Study site and experimental huts

The study was performed at the CREC/LSHTM experimental hut site in Cové, southern Benin (7°14′ N 2°18′ E). The station is situated at the centre of a large rice-growing zone which provides extensive mosquito breeding sites throughout the year. The rainy season extends from March to October and the dry season from November to February. The trial ran for 12 months from September 2016 to September 2017 in 9 experimental huts of the West African design [20]. The huts are built on concrete plinths surrounded by water-filled moats to prevent the entry of scavenging ants and are equipped with veranda traps to capture exiting mosquitoes. The walls are made of brick plastered with cement on the inside, with a corrugated steel roof, a ceiling of palm thatch and four window slits (1-cm gap) on the walls through which mosquitoes enter. The vector species consists of *Anopheles coluzzii* and *An gambiae* sensu stricto (*s.s*.) with the latter occurring at lower proportions (~ 23%) mostly in the dry season [21]. The vector population is very resistant to pyrethroids but susceptible to carbamates and organophosphates. Pyrethroid resistance is mediated by L1014 *kdr* (> 90% frequency) and over-expression of metabolic enzymes.

### WHO Susceptibility bioassays

To determine the frequency of insecticide resistance in the wild vector population during the hut trial, WHO cylinder bioassays were performed on 2–5-day old adult F1 female mosquitoes emerging from larvae collected from breeding sites close the experimental huts. Approximately 100 female mosquitoes per insecticide were exposed for 1 h in cohorts of 25 to alpha-cypermethrin 0.05%, permethrin 0.75%, deltamethrin 0.05% bendiocarb 0.1% and fenitrothion 1% treated filter papers. Knockdown was recorded after 1 h and mortality after a 24 h holding period.

### CDC bottle bioassays to investigate cross-resistance to chlorfenapyr

To investigate cross-resistance to chlorfenapyr and pyrethroids and determine a discriminating dose for the insecticide, CDC bottle bioassays were performed during the trial with 2-5 days old pyrethroid-resistant *An. gambiaes.l.* adult F1 female mosquitoes emerging from larvae collected from breeding sites close the experimental huts. Approximately 100 female mosquitoes per insecticide dose were exposed for 1 h in cohorts of 25 to bottles treated with chlorfenapyr at 5 different doses ranging from 15 to 450 µg/bottle. Mosquitoes were also exposed to untreated control and alpha-cypermethrin 12.5 µg treated bottles. Mosquitoes of the susceptible *An. gambiae* Kisumu strain were also tested for comparison. The tests were double-blind; Eppendorf tubes containing unknown pre-weighed amounts of the technical grade of each dose/insecticide obtained from BASF were used to prepare stock solutions and 1 ml of each was applied to each bottle.

### Experimental hut treatments

Three new formulations of chlorfenapyr coded FS1, FS7, and HKI and two new mixture formulations of alpha-cypermethrin and chlorfenapyr coded FS2 and F5 were compared to the reference Sylando^®^ 240SC, alpha-cypermethrin (FS4) and bendiocarb in the experimental hut trial at application rates provided in Table 1. The new formulations were selected based on their performance against susceptible and resistant strains of *An. gambiae s.l.* in preliminary WHO laboratory cone bioassays testing a wider range of formulations on mud and concrete substrates. Apart from HKI which was a wettable miscible granule formulation of chlorfenapyr, all other formulations tested in the huts were suspension concentrates (Table 1). The formulations were compared to bendiocarb WP and an untreated hut for the control.

**Table 1:** Experimental hut treatments and insecticide formulations

SN	Insecticide	Treatment code	Insecticide Formulation	Application rate
1	–	Control	–	–
2	Alpha-cypermethrin	FS4	Alpha 3.31% SC	30 mg/m^2^
3	Mixture (CFP + Alpha)	FS2	CFP 20.69% + Alpha 3.31% SC	250 mg/m^2^ + 30 mg/m^2^
4	Mixture (CFP + Alpha)	FS5	CFP 20.69% + Alpha 3.31% SC	250 mg/m^2^ + 30 mg/m^2^
5	Chlorfenapyr	FS1	CFP 20.69% SC	250 mg/m^2^
6	Chlorfenapyr	FS7	CFP 20.69% SC	250 mg/m^2^
7	Chlorfenapyr	Sylando 240 SC	CFP 24% SC	250 mg/m^2^
8	Chlorfenapyr	HKI	CFP 24.2% WMG	250 mg/m^2^
9	Bendiocarb	Bendiocarb	WP	400 mg/m^2^

*CFP* chlorfenapyr, *Alpha* alpha-cypermethrin, *SC* suspension concentrate, *WMG* wettable miscible granule

To prevent contamination from previous trials, hut walls were re-plastered and the concrete allowed to cure for a month before the trial. The IRS treatments were applied using a Hudson Xpert compression sprayer equipped with an 8002 flat fan nozzle following WHO guidelines [20]. To improve the accuracy of the spray application, hut walls were marked with spray swaths and a guidance pole attached to the tip of the spray lance to enable the spray man to maintain a fixed distance from the wall during spraying. Spray men wore full protective clothing and huts were sprayed from top to bottom using a predetermined lance speed to deliver the target volume. The palm thatch used to line the ceiling of the hut was sprayed flat on the ground outside the hut and allowed to dry before fitting to the ceiling of the hut. The volume sprayed was determined by subtracting the volume left in the tank after spraying. The actual volume sprayed on the walls did not deviate significantly from the target application rate for each hut (< 15% deviation for any treatment).

### Trial procedure and volunteer sleepers

The IRS treatments were applied on 16th September 2016 and the trial lasted for 12 months. Consenting human volunteer ‘sleepers’ slept in the huts from 9:00 p.m. to 5:00 a.m. each night to attract mosquitoes into the huts. To account for individual attractiveness to mosquitoes, sleepers were rotated through the huts daily using a simple Latin square design. At dawn, the volunteer sleepers collected dead mosquitoes in the room of the hut and all mosquitoes which escaped into the veranda using torches and aspirators. Mosquito collections were then transferred to the laboratory for processing where they were identified according to appropriate identification keys and scored for their blood-feeding status and mortality. Delayed mortality was recorded every 24 h for up to 72 h. Mosquitoes were held at 25 ± 2 °C during the observations.

### Outcome measures

The following outcome measures were used to assess the efficacy of the treatments in the experimental huts:

1. Deterrence: percentage reduction in the number of mosquitoes caught in treated hut relative to the number caught in the control hut
2. Exiting rates: due to potential irritant effect of treatments expressed as a percentage of the mosquitoes collected from the veranda trap
3. Blood feeding rate: percentage of blood-fed mosquitoes
4. Inhibition of blood-feeding: reduction in blood-feeding rate relative to the control. This was as follows:

$$100 \, (Bfu - Bft)/Bfu,$$

where Bfu is the proportion of blood-fed mosquitoes in the untreated control hut and Bft is the proportion of blood-fed mosquitoes in the hut with a specific insecticide treatment.

5. Mortality rate: percentage of dead mosquitoes after a 72 h holding period.

### Assessing spray quality

Before spraying, filter papers (Whatman No 1) measuring 5 × 5 cm were fixed on each wall in each hut using masking tape. After spraying, the filter papers were removed, carefully packaged in aluminium foil and stored at 4 °C for 10 days after which they were shipped to BASF for chemical analysis to assess the quality of the spray applications using gas chromatography.

### Wall cone bioassay exposure time studies

Supplementary bioassays were performed to investigate the relationship between exposure time in wall cone bioassays and mortality rates of free-flying mosquitoes. Fifty laboratory maintained susceptible female *An. gambiae* Kisumu mosquitoes per hut were exposed in cohorts of 10 for 30 min, 1 h, 2 h and 4 h to walls of huts treated with alpha-cypermethrin, Sylando 240SC and the control hut using standard WHO cone bioassays. One cone was placed on each wall and one on the ceiling. Mortality was recorded every 24 h for up to 72 h.

### Statistical analysis

Proportional outcomes (blood-feeding, exiting and mortality) related to each experimental hut treatment were assessed using binomial generalized linear models (GLMMs) on Stata (version 2.15.0). A separate model was fitted for each outcome. In addition to the fixed effect of each treatment, each model included random effects to account for the sources of variation between the volunteer sleepers and the months of the trial.

## Results

### WHO resistance bioassays

**Fig. 1:**
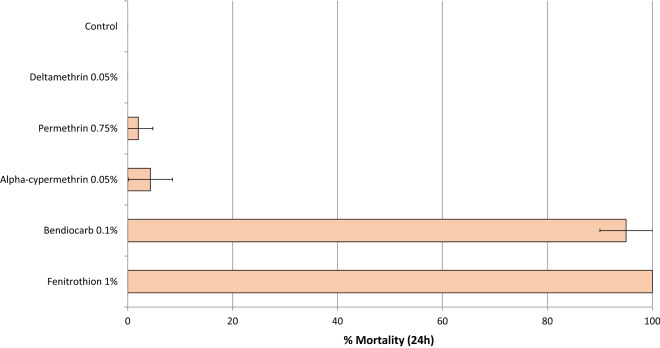
Mortality of wild pyrethroid resistant *Anopheles gambiae s.l.* from Cové in WHO susceptibility cylinder bioassays. Each bar represents mortality of ~ 100 exposed mosquitoes. Error bars represent 95% confidence intervals

Over 95% of wild *An. gambiae s.l.* mosquitoes from Cové survived exposure to pyrethroids (deltamethrin, permethrin and alpha-cypermethrin) in the WHO susceptibility cylinder bioassays thus confirming the high levels of pyrethroid resistance in the Cové vector population (Fig. 1). Mortality with bendiocarb 0.1% and fenitrothion 1% treated papers were 95% and 100%, respectively demonstrating susceptibility to carbamates and organophosphates.

### Cross-resistance CDC bottle bioassays

**Fig. 2:**
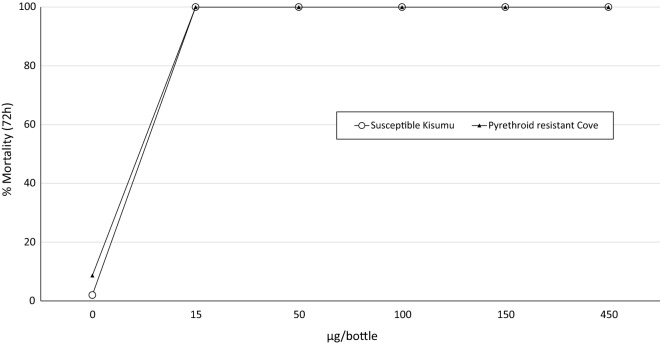
Mortality of susceptible *Anopheles gambiae* Kisumu and pyrethroid resistant *An. gambiae s.l.* Cové in chlorfenapyr treated CDC bottle bioassays

The mortality rate with alpha-cypermethrin 12.5 µg bottles was 48%. Mortality was 100% with all 5 doses of chlorfenapyr tested (15 to 450 µg) for both susceptible Kisumu and pyrethroid-resistant Cové strains (Fig. 2). There was no measurable difference in susceptibility to chlorfenapyr between both strains at the doses tested indicating no evidence of cross-resistance to chlorfenapyr in the wild pyrethroid-resistant vector population in Cové.

### Hut trial results

**Fig. 3:**
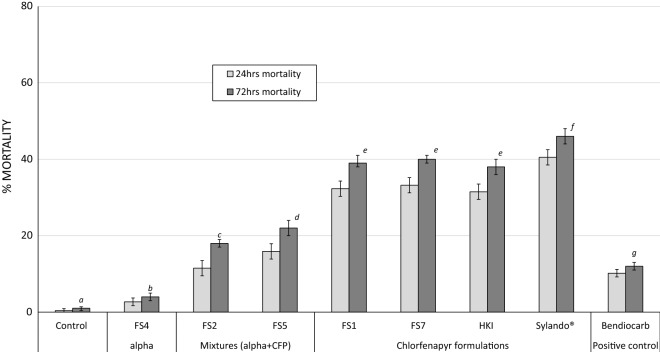
Overall mortality of wild free-flying pyrethroid resistant *Anopheles gambiae s.l.* Cové in IRS treated experimental huts in Cové, Benin. For 72 h mortality, bars bearing the same letter label are not significantly different at the 5% level (P > 0.05, logistic regression). Error bars represent 95% confidence intervals. *CFP* chlorfenapyr

**Fig. 4:**
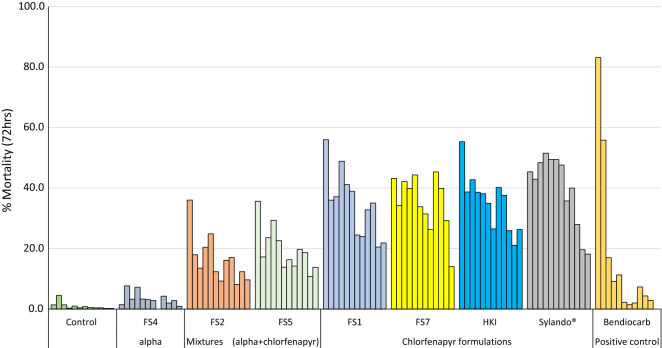
Monthly mortality of wild free-flying pyrethroid resistant *Anopheles gambiae s.l.* entering IRS treated experimental huts in Cové Benin. Error bars represent 95% confidence intervals

The hut trial results are presented in Table 2 and Figs. 3 and 4. A total of 32,486 female *An. gambiae s.l.* were collected in the experimental huts during the trial. The exiting rates in huts treated with chlorfenapyr formulations (71–80%) were lower than what was observed in huts treated with alpha-cypermethrin alone (FS4), the mixture formulations (FS2 and FS5) and bendiocarb (94–100%, P < 0.05). This can be attributed to the high excito-repellent and neurotoxic activities of the pyrethroid and the carbamate. Blood-feeding rates were 100% with the untreated control and 98–100% with all treatments tested. This is expected of the IRS treatments considering there was no mosquito net barrier to prevent feeding, and that mosquitoes feed on sleepers before resting on the treated walls.

**Table 2:** Results with wild free-flying pyrethroid-resistant *An. gambiae* s.l. mosquitoes entering IRS treated experimental huts in Cové, Benin

Hut treatments	Control	Alpha alone	Alpha + chlorfenapyr mixture formulations		Chlorfenapyr formulations				Positive control
	Untreated hut	FS4	FS2	FS5	FS1	FS7	HKI	Sylando^®^ SC	Bendiocarb
Females caught	6167	3488	4350	3383	3153	2563	2784	3187	3411
% Deterrence	–	43	29	45	49	58	55	48	45
% Exophily	50	100	98	94	80	79	78	71	96
95% conf. limits	48–51	99–100	97–98	93–95	79–81	77–80	77–80	69–72	96–97
N blood-fed	6141	3455	4325	3365	3079	2492	2771	3154	3353
% Blood fed	100	99	99	99	98	97	100	99	98
95% conf. limits	99–100	98–100	99–100	99–100	97–98	96–98	99–100	99–100	97–100
N dead 72 h	50	144	782	746	1243	1028	1044	1462	432
% Overall 72 h mortality*	1^a^	4^b^	18^c^	22^d^	39^e^	40^e^	38^e^	46^f^	12^g^
95% conf. limits	0–2	3–5	17–19	21–23	38–41	38–42	36–39	44–48	11–13

*Values along this row bearing the same letter superscript are not significantly different (P > 0.05, logistic regression)

Overall mortality through the course of the 12-month trial was 1% in the control hut and 4% in the alpha-cypermethrin treated hut (FS4). The chlorfenapyr and alpha-cypermethrin mixture formulations induced lower levels of overall mortality compared to what was achieved with the chlorfenapyr-only formulations (18–22% vs. 38–46% P < 0.05). Overall mortality was much lower with bendiocarb (12%) compared to chlorfenapyr alone (38% to 46%, P < 0.05) and mixture formulations (18–22%, P < 0.05). The highest mortality was achieved with the Sylando^®^ 240SC formulation and this was significantly higher than what was achieved with other chlorfenapyr-only formulations (46% with Sylando^®^ 240SC vs. 38–40% with other chlorfenapyr-only formulations, P < 0.05). There was evidence of delayed mosquito mortality with the mixtures and chlorfenapyr formulations as mortality increased significantly with these treatments when observation time increased from 24 to 72 h; this effect was not observed with alpha-cypermethrin alone and bendiocarb (Fig. 3).

#### Monthly mortality of wild mosquitoes in treated experimental huts

The monthly mortality rates of wild free-flying pyrethroid-resistant *An. gambiae s.l.* entering the IRS treated huts in Cové over the 12-month trial are presented in Fig. 4. Initial mortality with the chlorfenapyr formulations was ~ 44–56% in the first month of the trial. This mortality remained more or less steady from month 2 onwards only dropping to ~ 20–30% after 8–10 months. By contrast, mortality with bendiocarb was initially > 80% in month 1 but declined very sharply to less than 20% by the third month. With the Sylando^®^ 240SC chlorfenapyr formulation, mortality was 40–50% for up to 9 months and dropped to 20% after 10 months.

#### Impact of exposure time in cone bioassays on IRS treated walls

**Fig. 5:**
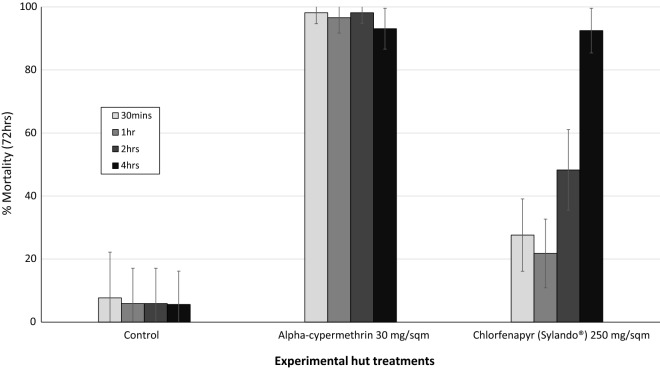
Cone bioassay mortality of susceptible *Anopheles gambiae* Kisumu exposed for a range of exposure times to IRS treated experimental hut walls 1 week post-treatment. Error bars represent 95% confidence intervals

Cone bioassay mortality rates of susceptible *An. gambiae* Kisumu mosquitoes exposed to alpha-cypermethrin and chlorfenapyr (Sylando^®^) treated hut walls for a range of exposure times 1 week post-IRS application are presented in Fig. 5. Mortality with alpha-cypermethrin was > 90% independent of exposure time. With chlorfenapyr, mortality was very low at the standard 30-min exposure time (27%) but increased steadily as exposure time was increased to 4 h. A mortality rate of 40–50% which was more consistent with the mortality of free-flying wild mosquitoes in the huts was only achieved after 2 h of exposure. Optimal cone bioassay mortality on chlorfenapyr treated walls (> 90% mortality) required 4 h of exposure.

#### Assessing spray quality

The results from chemical analysis of filter papers that were fixed to hut walls during spraying are presented in Table 3. Apart from the FS5 which showed a deviation from target dose of 51%, the mean insecticide content of the filter papers were generally within the acceptable deviation of less than 50% [22] ranging between 160 and 299 mg/m^2^ for chlorfenapyr (target was 250 mg/m^2^) and 18 mg/m^2^ to 41 mg/m^2^ for alpha-cypermethrin alone (target was 30 mg/m^2^).

**Table 3:** Chemical analysis of filter papers from IRS-treated experimental huts in Cové, Benin

Hut treatment	Chlorfenapyr content (mg/m^2^)	% Deviation	Alpha content (mg/m^2^)	% Deviation
FS1	299	19	–	–
FS2	271	9	41	28
FS4	–	–	21	30
FS5	122	− 51	18	40
FS7	242	− 3	–	–
Sylando^®^	164	− 34	–	–
HKI	160	− 36	–	–

Mean insecticide content

## Discussion

New chemical compounds with novel modes of action which can provide both improved and long-lasting residual control of insecticide-resistant malaria vector populations are urgently needed for indoor residual spraying [1]. The effectiveness and residual activity of IRS often depend on the type of formulation of the insecticide used. In this study, we evaluated the efficacy of several formulations of chlorfenapyr alone and as mixtures with alpha-cypermethrin in the controlled household environment of an experimental hut study against a naturally entering and exiting pyrethroid-resistant vector population in an attempt to identify a more effective and longer-lasting formulation of the insecticide for IRS than the reference Sylando 240 SC chlorfenapyr formulation. The selection of formulations for testing was based on their good performance in preliminary laboratory bioassays of a wide range of WG, WP, SC and novel formulations of chlorfenapyr on mud and concrete substrates. IRS with the carbamate bendiocarb and pyrethroid alpha-cypermethrin served as positive controls as these have been used in West Africa.

The low overall mortality of free-flying mosquitoes in huts treated with alpha-cypermethrin IRS (4%) demonstrates the ineffectiveness of pyrethroids for IRS in areas with high levels of pyrethroid resistance and highlights the need for effective non-pyrethroid IRS insecticides. Sylando 240SC formulation of chlorfenapyr induced a mean mortality rate over 12 months of 46% thus confirming results from previous experimental hut studies [16, 17]. However, contrary to expectations, none of the new chlorfenapyr and mixture formulations tested was an improvement over Sylando 240SC in the experimental huts thus demonstrating a major challenge in developing an improved more effective formulation of chlorfenapyr based on SC or WDP technology for IRS. Throughout the trial, all the chlorfenapyr solo and mixture formulations tested induced much higher overall mortality compared to what was achieved with bendiocarb, a WHO-recommended IRS insecticide to which the vector population was fully susceptible, thus showing that chlorfenapyr is nonetheless a potentially effective insecticide for IRS.

To ensure that householders in areas with stable malaria transmission are protected by IRS, it is desirable that the insecticide deployed persists long enough on treated home walls to cover the entire transmission season, otherwise multiple and resource-demanding IRS applications may be required [23]. Initial mortality with bendiocarb was 80% but this lasted for only 1 month and was almost completely lost by the third month of the trial, thus confirming its suitability only for short transmission seasons on concrete wall substrates [24]. By contrast, the current study demonstrated the long residual activity of chlorfenapyr (Sylando 240SC) on treated concrete walls killing free-flying pyrethroid-resistant malaria vectors substantially for up to 9 months. The mortality rate was lower than the initial mortality rate in huts treated with bendiocarb but was persistent throughout the study and never declined as observed in the bendiocarb treated huts. This pattern is unusual and raises the question of what level of mortality as demonstrated in an experimental hut is required to provide adequate transmission control on a community scale. New classes of chemistry, particularly those that have a unique, non-neural mode of action are required to undergo cluster randomised trials if they are applied for an insecticide-treated net [25], but this is not a strict requirement for an IRS. Nevertheless, the question of what level of control in an experimental hut would translate to transmission control is a valid one which requires better evidence. The question of who should pay to obtain this evidence also remains open.

While higher levels of mortality of pyrethroid-resistant vector populations have been reported with other recently developed novel IRS insecticides in similar trials [7, 13–15], effective rotation of IRS insecticides for insecticide resistance management as recommended by the GPIRM will require a much larger and more diversified portfolio of IRS insecticides [2].

Considering its unique mode of action and long residual activity against pyrethroid-resistant mosquitoes, chlorfenapyr has the potential to efficiently complement other IRS compounds in an IRS rotation programme for insecticide resistance management. In addition, chlorfenapyr IRS has shown potential to complement standard pyrethroid only nets in a combined intervention strategy against pyrethroid-resistant malaria vectors inducing an additive effect of both interventions [16, 26]. It is also unknown whether chlorfenapyr, with its unique mode of action, has other effects on the vector not apparent in experimental huts.

The lower mortality rates achieved with the alpha-cypermethrin and chlorfenapyr IRS mixture formulations compared to the chlorfenapyr solo IRS formulations was consistent with findings from a previous study which evaluated a tank mix of alphacypermethrin SC (Fendona SC) and chlorfenapyr SC (Sylando SC) formulations against the same vector population [17]. This effect in the previous study might have been due to the carriers in the two formulations masking or covering one another. The alternative hypothesis is that high excito-repellent property of the pyrethroid may have prevented adequate contact with the more toxic chlorfenapyr component of the mixture to allow pick-up of a toxic dose from the treated walls. Because this trend with the co-formulated mixture products in the present study was consistent with that shown in the earlier tank-mix trial, the neutralising effect on chlorfenapyr toxicity was, therefore, more likely due to excito-repellency of the pyrethroid in the co-formulated mixtures than on masking by the constituent SC carrier formulants. Based on this observation, the manufacturing company (BASF) decided to discontinue development of the mixture formulation for IRS.

While the percentage mortality of free-flying mosquitoes that enter experimental huts and alight on walls before or after blood-feeding is the primary measure of the efficacy of IRS in WHO phase 2 experimental hut trials, the standard WHO technique for measuring and monitoring the bioefficacy and residual activity of applied IRS is the 30 min cone bioassay on wall surfaces. Previous studies with chlorfenapyr IRS have demonstrated the inadequacy of 30 min exposure to predict the performance of chlorfenapyr IRS against free-flying mosquitoes [17, 27]. The present study demonstrated that with chlorfenapyr, wall cone bioassays may only predict mortality of free-flying mosquitoes if the mosquito exposure time is increased to 2–4 h. Two-hour exposure may be the average exposure time free-flying mosquitoes spend on chlorfenapyr IRS surfaces. Chlorfenapyr treated surfaces are non-repellent unlike alpha-cypermethrin surfaces [28] where 30 min is established as a more appropriate exposure time. This raises the intriguing prospect of insecticide-class specific exposure times. Previous research on pirimiphos methyl-treated surfaces indicates a 1 h exposure is the more appropriate and realistic exposure time for organophosphate that predicts mortality of free-flying mosquitoes [7]. Organophosphates are more repellent than chlorfenapyr but less repellent than pyrethroids such as alpha-cypermethrin [29]. Insecticide class-specific exposure time may prove to be an advance on current practice and deserves further attention. Research on chlorfenapyr is further complicated by its non-neurotoxic mode of action on mitochondrial respiratory pathways, which is most evident in host-seeking and flying mosquitoes. It may be the case that blood-fed mosquitoes resting on chlorfenapyr treated walls are less sensitive to the toxicity of the insecticide than free-flying, unfed, host seeking mosquitoes approaching an alpha-cypermethrin and chlorfenapyr mixture LLIN (Interceptor^®^ G2) [16]. This observation further highlights the need for new testing methods and guidelines, which take into consideration the mode of action of novel non-neurotoxic insecticides.

## Conclusion

Indoor residual spraying with chlorfenapyr provides moderate but prolonged control of pyrethroid-resistant malaria vectors compared to pyrethroid and bendiocarb IRS. The Sylando 240SC formulation of chlorfenapyr outperformed all other newly developed formulations indicating that the level of mortality achieved was not improved by reformulation. New formulations of mixtures of chlorfenapyr and alpha-cypermethrin did not improve the bioefficacy of the chlorfenapyr IRS alone and confirmed the negative effect of alpha-cypermethrin in the IRS mixture. Wall cone bioassays on chlorfenapyr treated walls required longer exposure times of 2 h than the customary 30 min stipulated by the WHO to generate data more representative of chlorfenapyr IRS bioefficacy in experimental huts. WHO guidelines on residual cone bioassays need to be more insecticide-specific.

## Data Availability

The datasets used and/or analysed during the current study are available from the corresponding author on reasonable request.

## Abbreviations

IRS: Indoor Residual Spraying
WHO: World Health Organization
PQ: prequalification team
GPIRM: Global Plan for Insecticide Resistance Management
WHOPES: WHO Pesticide Evaluation Scheme
CREC: Centre de Recherche Entomologique de Cotonou
LSHTM: London School of Hygiene & Tropical Medicine
Kdr: knockdown resistance
